# Expression and potential role of cellular retinol binding protein I in psoriasis

**DOI:** 10.18632/oncotarget.26314

**Published:** 2018-12-04

**Authors:** Gaetana Costanza, Elena Doldo, Amedeo Ferlosio, Chiara Tarquini, Daniela Passeri, Raffaella Cascella, Mauro Bavetta, Alessandro Di Stefani, Claudio Bonifati, Sara Agostinelli, Federica Centofanti, Emiliano Giardina, Elena Campione, Luca Bianchi, Pietro Donati, Aldo Morrone, Augusto Orlandi

**Affiliations:** ^1^ Anatomic Pathology Unit, Department of Biomedicine and Prevention, Tor Vergata University of Rome, Rome, Italy; ^2^ Genetics Laboratory, Department of Biomedicine and Prevention, Tor Vergata University of Rome, Rome, Italy; ^3^ Dermatology Unit, Tor Vergata University of Rome, Policlinic of Tor Vergata of Rome, Rome, Italy; ^4^ San Gallicano Dermatology Institute, Rome, Italy; ^5^ Catholic University “Our Lady of Good Counsel”, Tirana, Albania

**Keywords:** CRBPI, retinoids, imiquimod-induced psoriasis, keratinocyte proliferation

## Abstract

Psoriasis is a diffuse chronic skin disorder characterized from accelerated epidermal turnover and inflammatory cell infiltrate. Retinoids influence keratinocyte proliferation and differentiation as well as inflammatory response. Cellular retinol binding protein (CRBPI) regulates intracellular vitamin A bioavailability and contributes to maintain skin homeostasis. The aim of present study was to investigate the expression of CRBPI and its role in the pathogenesis of skin psoriasis. Immunohistochemistry revealed more diffuse and increased CRBPI expression in all epidermal layers of human psoriatic lesions except in the stratum corneum. An imiquimod-induced psoriatic-like model documented the increase of skin lesional area and severity index score as well as of the severity of microscopic features as parakeratosis, papillomatosis and spongiosis in CRBPI-knockout compared to wild-type mice, associated to the increased keratinocyte CK17 and Ki-67 expression and the reduction of CK1, CRABPII and RXRα. Gene array of imiquimod-induced psoriatic skin documented the greater up-regulation of EGF/PDGF-related genes and down-regulation of EGR1 and pro-inflammatory IL-related genes in CRBPI-knockout compared to wild-type mice. Finally, CRBPI transfection in HaCaT cells increased AKT and NF-κB-related genes and proteins and down-regulated IL-2, IL-6 and IL-8 pro-inflammatory signalling. Although not recognized as a psoriatic susceptibility gene in our cohort of patients, the present data strongly supported the potential role of CRBPI to sustain keratinocyte proliferation and differentiation and to counteract pro-inflammatory genes expression in psoriatic lesions.

## INTRODUCTION

Psoriasis is one of the most frequent skin disorders, affecting about 2-3% of the general population [1]. Psoriasis represents a life-long patients’ affliction, with a considerable impact on the quality of life [2, 3]. Psoriasis is characterized by epidermal hyperproliferation, abnormal terminal keratinocyte differentiation and inflammation [4]. Risk and genetic factors interact with environmental agents such as drugs, stress or infections [1, 3, 5, 6] but still many aspects of the pathogenesis of psoriasis remain partially unknown. Retinoids regulate proliferation and differentiation of skin keratinocytes [7]. Cellular retinol binding protein I (CRBPI) is a cytosolic carrier of retinol essential for its homeostasis [8–10]. CRBPI expression is indispensable for embryonic development, growth and survival of vertebrates [9]. In the skin, CRBPI contributes to keratinocyte growth and differentiation likely by regulating intracellular retinol bioavailability [11]. Altered expression of retinoid-related proteins and receptors have been reported in the psoriatic skin [12–14], but data concerning CRBPI expression were controversial [13, 15]. In fact, first studies on potential CRBPI involvement in psoriasis showed its decreased expression in psoriatic compared to non-lesional skin both in basal condition and after retinoids treatment, with a relevant inter-individual variability [15]. Successively, Bush et al., [12] reported an increased CRBPI expression in psoriasis. In another study, the amount of CRBPI protein was reported to be similar in non-lesional and lesional psoriatic skin cells [13]. CRBPI gene (*RBP1*) maps on chromosome 3q2 and clusters near PSORS5 locus, part of psoriasis susceptibility *loci* [16]. In the present study, we investigated CRBPI expression in lesional and non-lesional skin of a cohort of patients affected by moderate to severe plaque type psoriasis. To better investigate the effective role of CRBPI expression in psoriatic lesion development and keratinocyte biology, we used an imiquimod-induced model of psoriasis [8, 17] in CRBPI-knockout mice and CRBPI-transfected HaCaT cells. Our results strongly support a favorable role of CRBPI expression in the maintenance of skin integrity in psoriasis by sustaining keratinocytes proliferation, differentiation and the expression of pro-inflammatory cytokines, suggesting CRBPI-mediated function a therapeutic target to prevent epidermal damage in psoriasis.

## RESULTS

### CRBPI overexpression is characteristic of psoriatic epidermis

**Figure 1:**
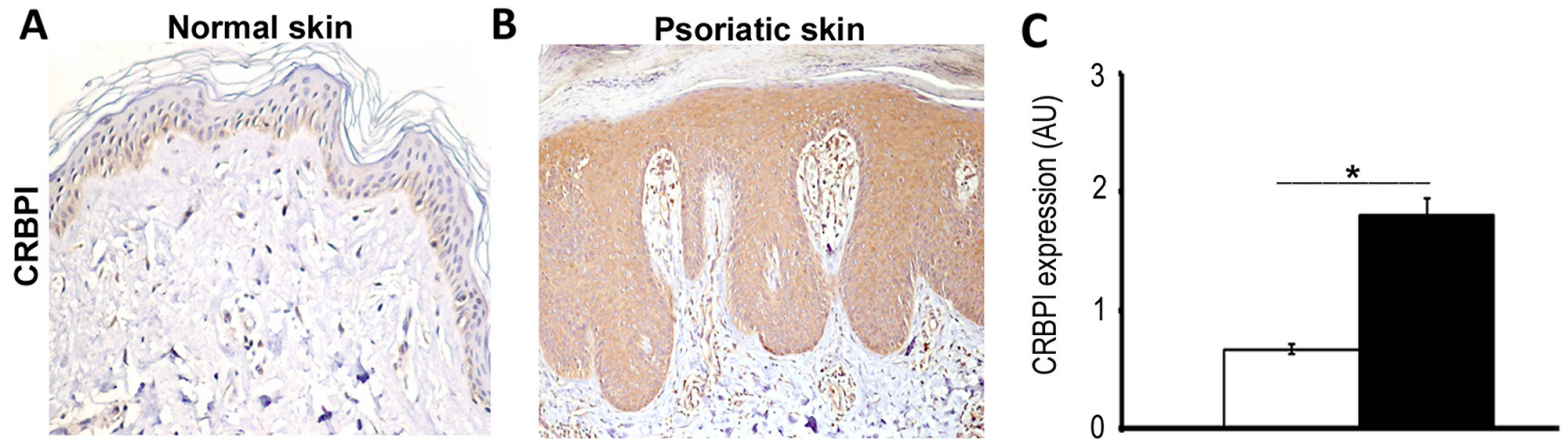
Expression of CRBPI in normal and psoriatic skin **(A)** Representative image of the weak CRBPI immunoexpression, more in the basal layer of the normal skin epidermis; whereas **(B)** CRBPI expression strongly increases in psoriatic epidermis. **(C)** Bar graph shows the semiquantitative evaluation of epidermal CRBPI immunostaining. Results are expressed as mean values±SEM. ^*^p<0.00001 at Student’s *t*-test.

We investigated CRBPI expression in human skin by immunohistochemistry. In normal epidermis, keratinocyte CRBPI immunodetection was faint and mostly cytoplasmatic, stronger in the basal than in superficial layers and absent in the corneum (Figure 1A). In psoriatic lesions, CRBPI immunoexpression was more diffuse increased compared to normal skin (p<0.00001 Figure 1B, 1C). CRBPI expression was absent in psoriatic parakeratotic cells. We also evaluated other parameters involved in the aberrant keratinocyte proliferation/differentiation of psoriasis. As reported in Supplementary Figure 1, epidermal Ki-67 and CK17 immunoexpression markedly increased in psoriasis compared to normal skin, (^**^p< 0.01) according to previous studies [3], whereas RXRα and CK1 expression decreased (^**^p<0.05; Supplementary Figure 1). Finally, epidermal CRABPII expression was also increased in psoriatic lesions compared to normal skin (^**^p< 0.01; Supplementary Figure 1).

### Imiquimod-induced skin inflammation is more severe in CRBPI-knockout mice

**Figure 2:**
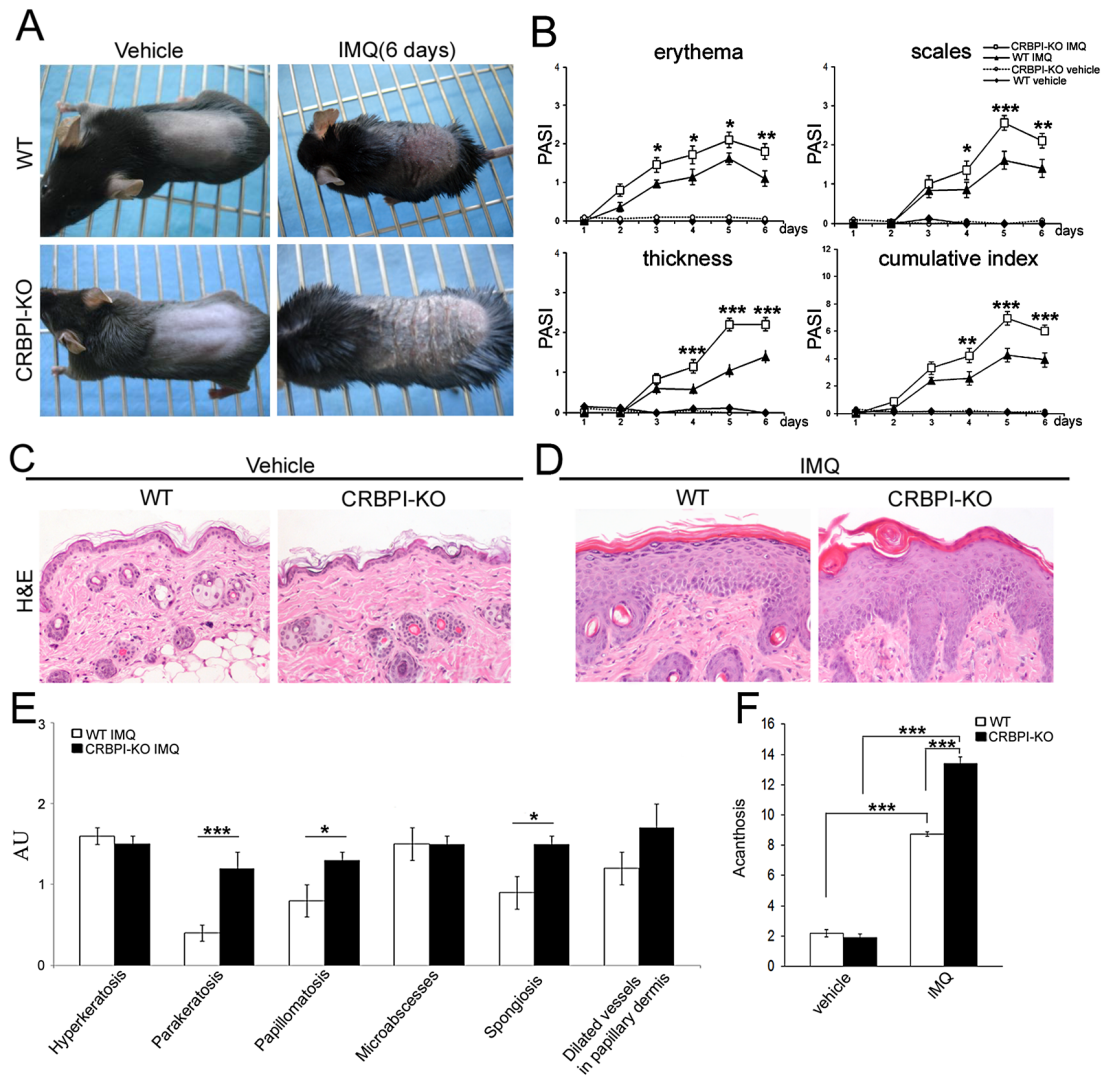
Imiquimod treatment induces more severe psoriasis-like lesions in CRBPI-knockout mice **(A)** Back skin of wild-type (WT) and CRBPI-knockout (KO) mice after 6 days of treated control cream (vehicle) or imiquimod (IMQ); **(B)** daily scoring of erythema, scaling and thickness cumulative score (PASI) shows an early increase in imiquimod treated CRBPI-knockout skin compared to wild-type mice; **(C-D)** representative microscopic images of (C) vehicle control and (D) imiquimod-treated wild-type and CRBPI-knockout mouse skin, Haematoxylin&Eosin (H&E) staining; original magnification; 200X; **(E-F)** bar graphs showing semiquantitative evaluation of microscopic parameters of skin psoriasis in imiquimod-treated wild-type and CRBPI-knockout mice after six days. Results are expressed as mean values±SEM (n=8 mice per group); ^*^p < 0.05; ^**^p < 0.01;^***^p < 0.001 at Student’s *t*-test. Original magnification, 200X.

To define a potential protective or favouring role of CRBPI in the development of psoriatic lesions, we investigated imiquimod-induced back skin inflammation, a well-known experimental model of psoriasis [17, 18] induced in CRBPI-knockout and wild-type C57BL/6 mice. After six days of imiquimod treatment, both groups showed typical skin signs of psoriasis (Figure 2A). Clinical evaluation of parameters such as erythema, scales and epidermal thickness revealed the greater increase in CRBPI-knockout compared to wild-type mice starting from days 2-3 (^*^p< 0.05; ^**^p< 0.01;^***^p< 0.001; respectively; Figure 2B). Also the cumulative psoriasis area and severity index (PASI) score was greater in CRBPI-knockout compared to wild-type mice (Figure 2B). Vehicle-treated skin of both mouse phenotypes as control did not reveal any sign of inflammation. Microscopic evaluation of Haematoxylin&Eosin-stained sections showed in untreated wild-type and CRBPI-knockout epidermis the presence of thin basal and granular layers and a more evident stratum corneum (Figure 2C). After 6 days of imiquimod treatment, semiquantitative analysis revealed that parakeratosis, papillomatosis and spongiosis were more severe in CRBPI-knockout compared to wild-type mice (^*^p< 0.05;^**^p< 0.01;^***^p<0.001; Figure 2D). Moreover, the number of epidermal layers was greater in CRBPI-knockout compared to wild-type mice (^***^p<0.001; Figure 2F). In summary, imiquimod induced more severe psoriasis-like skin lesions in CRBPI-knockout than in wild-type mice.

### Expression of CRABPII, RXRα and other psoriasis biomarkers is decreased in CRBPI-knockout mice

**Figure 3:**
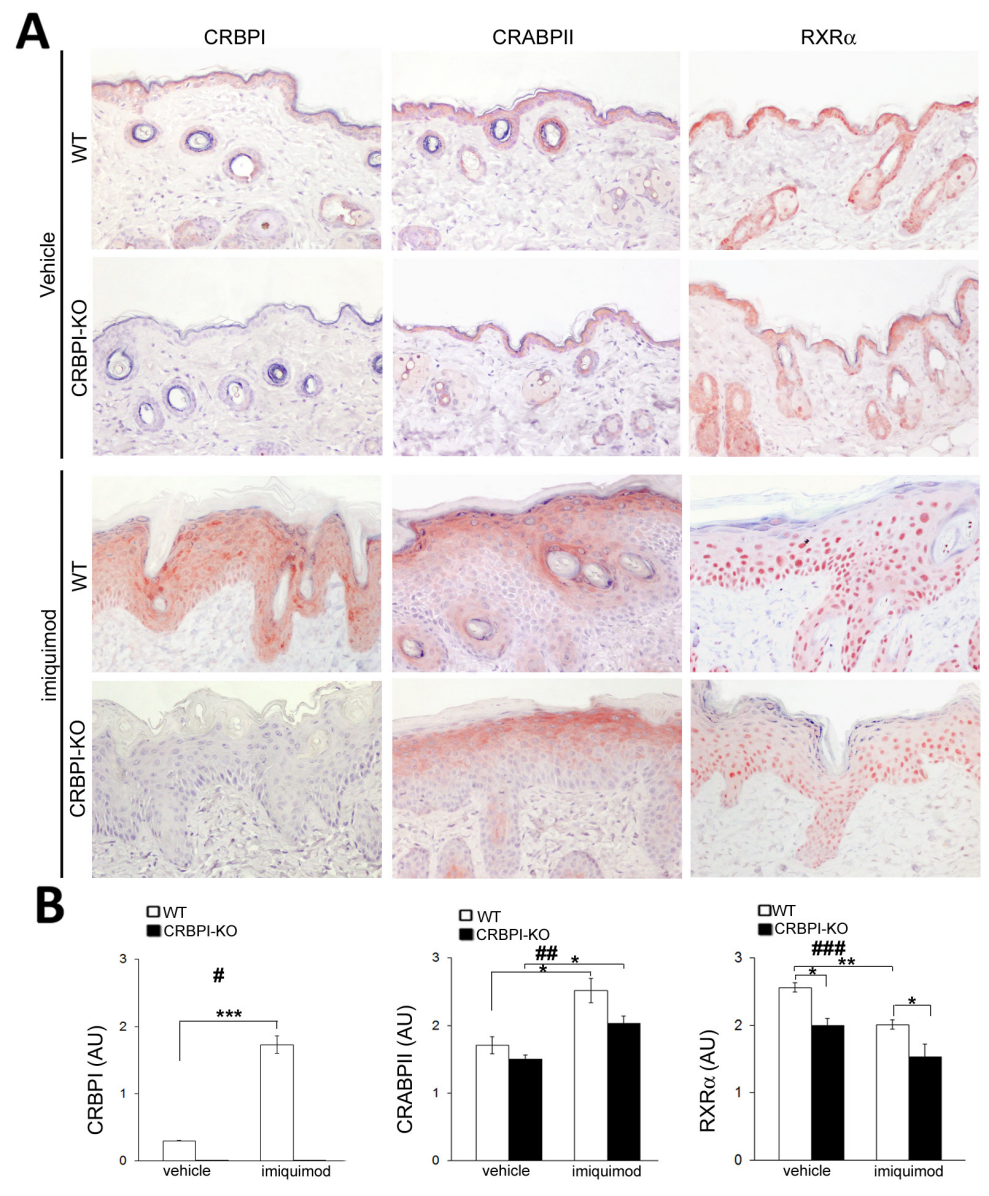
Evaluation of CRBPI, CRABPII and RXRα expression in imiquimod-induced psoriatic skin of CRBPI-knockout and wild-type mice **(A)** Representative CRBPI, CRABP-II and RXRα immunostainings of wild-type (WT) and CRBPI-knockout (KO) mice skin after 6 days of treatment with control cream (vehicle) or imiquimod (IMQ). Original magnification 200X; **(B)** Bar graphs show the semiquantitative evaluation of CRBPI, CRABPII and RXRα immunostainings. Results are expressed as mean values±SEM (n=8 mice per group); ANOVA ^#^*p* < 0.005, ^##^*p* < 0.0005, ^###^*p* < 0.000005. ^*^p < 0.05; ^**^p < 0.01;^***^p< 0.001 at Student’s *t*-test.

**Figure 4:**
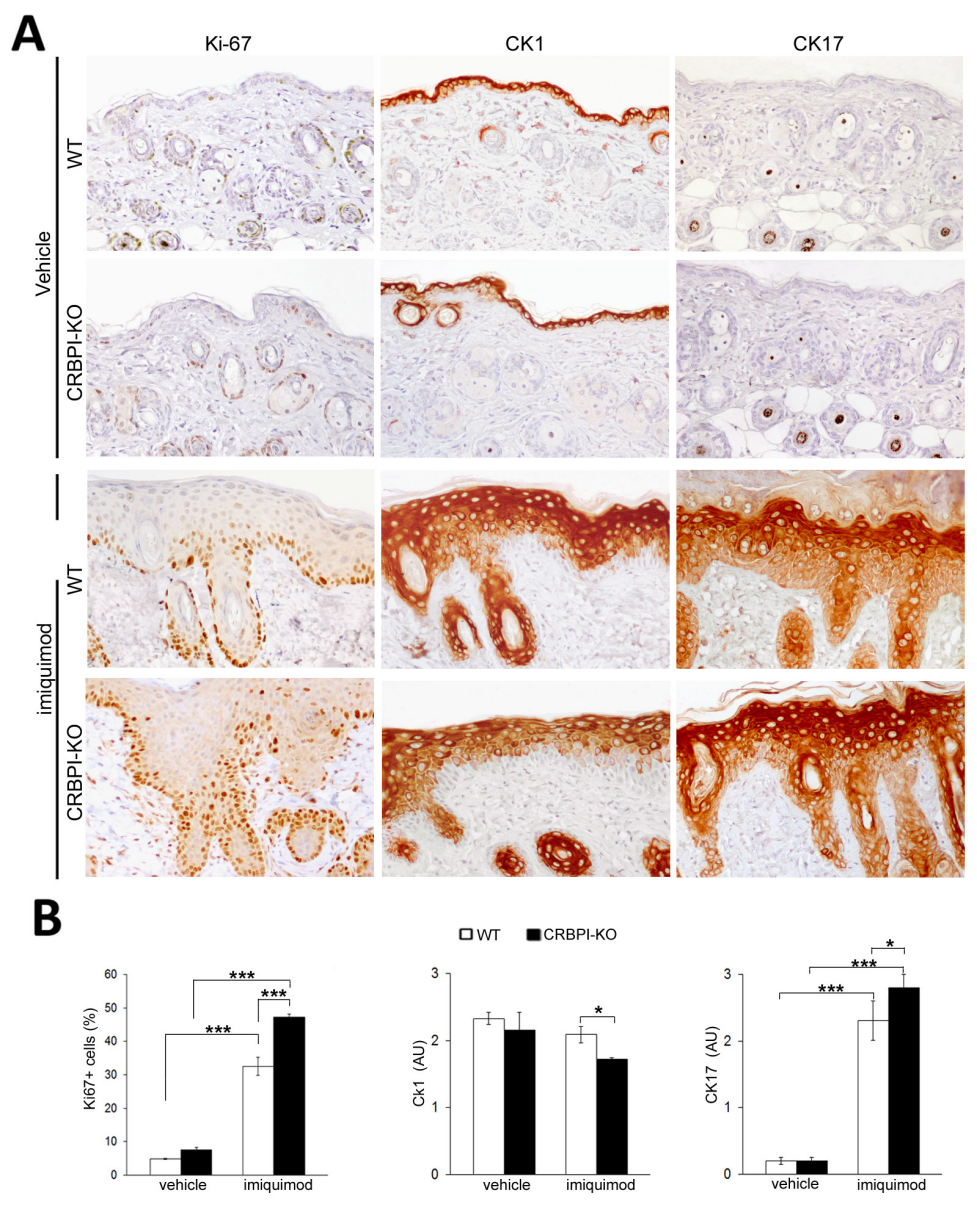
CRBPI gene loss influences epidermal proliferation and expression of differentiation markers **(A)** Representative Ki-67, CK1 and CK7 immunostainings of wild-type (WT) and CRBPI-knockout (KO) back skin after 6 days of daily treatment with control cream (vehicle) or imiquimod (IMQ); **(B)** bar graphs showing the semiquantitative evaluation of Ki-67, CK1 and CK17 immunostainings; results are expressed as mean values±SEM (8 mice per group); ANOVA ^#^*p* < 0.05, ^##^*p* < 0.0005, ^###^*p* < 0.000005. ^*^p< 0.05; ^**^p < 0.01;^***^p< 0.001 at Student’s *t*-test.

We investigated the expression of retinoid receptors and psoriatic biomarkers in normal and imiquimod-treated mouse skin. CRBPI immunostaining detected in the skin of wild-type C57BL/6 mice but not in CRBPI-knockout mice, as expected (Figure 3A). After six days of imiquimod treatment, CRBPI expression increased in the epidermis of wild-type compared to untreated control mice (p<0.0001, Figure 3B), and still absent in CRBPI-knockout mice. Interestingly, we documented a reduced CRABPII and RXRα epidermal expression in imiquimod-treated CRBPI-knockout compared to wild-type mice (Figure 3A and 3B). As reported in Figure 4A and 4B, epidermis of vehicle-treated wild-type and CRBPI-knockout mice displayed a similar Ki-67, CK1 and CK17 immunopositivity. After 6 days of imiquimod treatment, the increase of Ki-67 and CK17 positivity and the reduction of CK1 expression were more evident in the epidermis of CRBPI-knockout compared to wild-type mice (Figure 4A and 4B). These results strongly supported the hypothesis that loss of CRBPI gene favors the severity of psoriatic lesions and negatively influences CRABPII and RXRα expression.

### CRBPI loss influences EGF/PDGF-related gene levels *in vivo*

**Figure 5:**
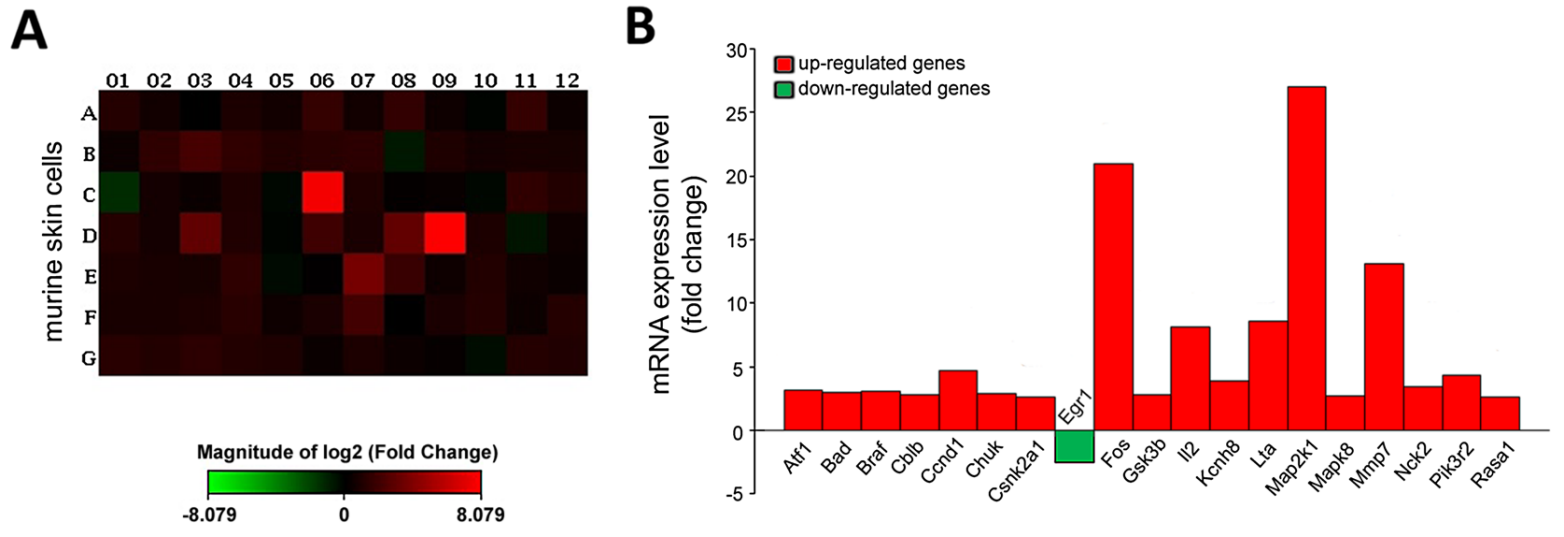
CRBPI deletion influences gene signaling in imiquimod-treated murine skin cells **(A)** Heat map and **(B)** bar graph of RT^2^ profiler™ PCR assay of EGF/PDGF-related genes in isolated murine skin of imiquimod-treated wild type and CRBPI-knockout mice. Up-regulated and down-regulated genes reported in red and green, respectively.

To better investigate gene pathways influenced by CRBPI expression *in vivo*, we performed gene array of imiquimod-treated skin of wild type and CRBPI-knockout mice. As shown in Figure 5A and 5B, imiquimod-induced psoriatic skin inflammation in CRBPI-knockout mice associated with a greater up-regulation of the following genes: activating transcription factor 1 (ATF1), BCL2-associated agonist of cell death (BAD), Braf transforming gene (BRAF), Casitas B-lineage lymphoma (CBLB), cyclin D1 (CCND1), conserved helix-loop-helix ubiquitous kinase (CHUK), casein kinase 2, alpha 1 polypeptide (CSNK2A1), FBJ osteosarcoma oncogene (FOS), glycogen synthase kinase 3 beta (GSK3B), interleukin 2 (IL-2), potassium voltage-gated channel, subfamily H (eag-related) member 8 (KCNH8), lymphotoxin A (LTA), mitogen-activated protein kinase kinase 1 (MAP2K1), mitogen-activated protein kinase 8 (MAPK8), matrix metallopeptidase 7 (MMP7), non-catalytic region of tyrosine kinase adaptor protein 2 (NCK2), phosphatidylinositol 3-kinase, regulatory subunit, polypeptide 2 (p85 beta) (PIK3R2), RAS p21 protein activator 1 (RASA1). Instead, early growth response 1 (EGR1) was down-regulated. Overall, our results suggested that loss of CRBPI expression influences epidermal growth factor-platelet-derived growth factor (EGF/PDGF)-related genes level, likely involved in keratinocyte growth and differentiation and down-regulated EGR1, involved in the control of keratinocyte hyperproliferation.

### CRBPI transfection restores AKT and NF-kB-related gene and protein levels in HaCaT cells

**Figure 6:**
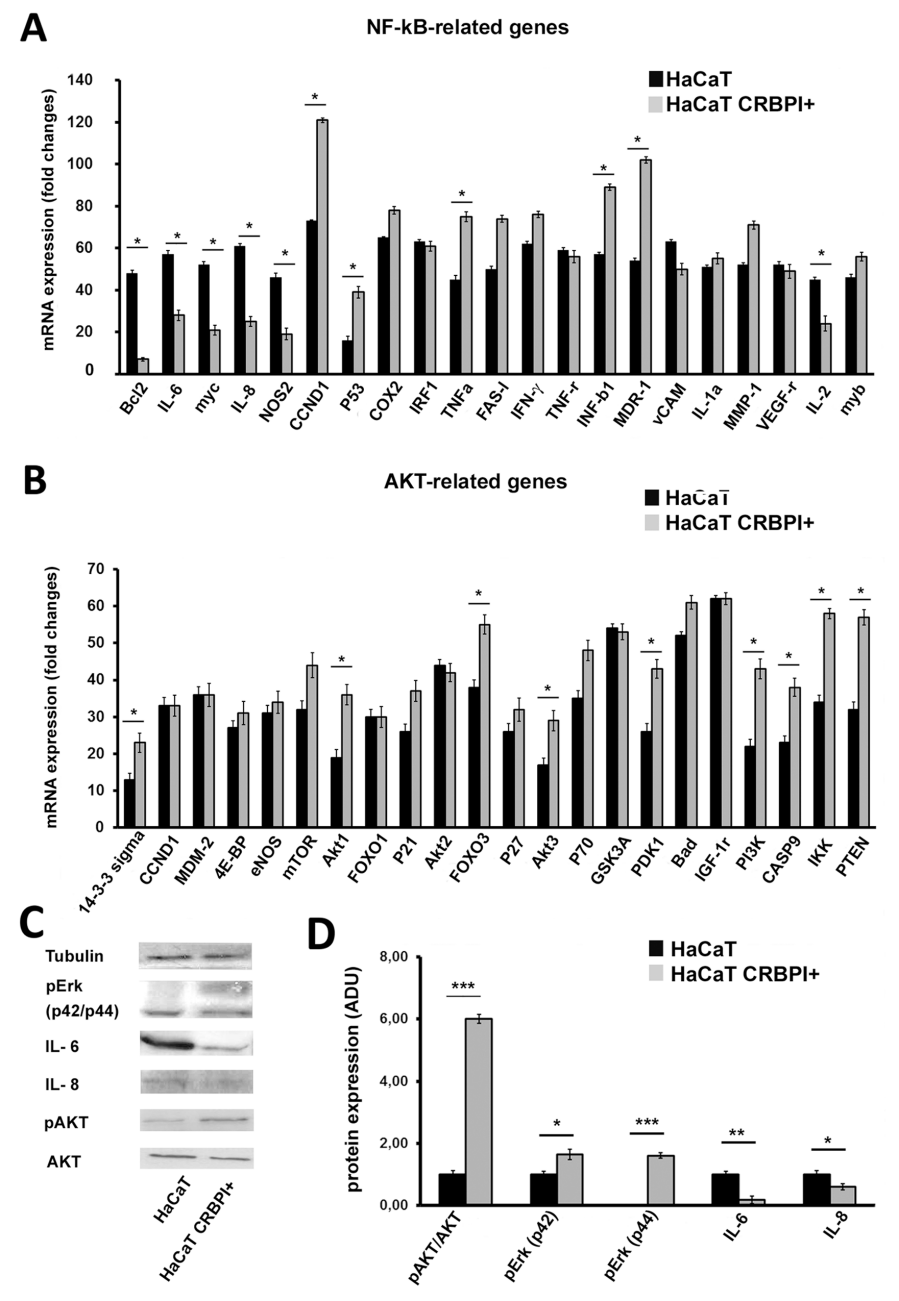
CRBPI transfection up-regulates NF-kB and AKT-related transcriptional gene pathway in HaCaT cells **(A-B)**, bar graphs of NF-kB and AKT signaling-specific genes array of CRBPI-transfected HaCaT cells. **(C)** Representative blots and **(D)** bar graphs densitometric evaluation of pAKT/AKT, pErk, IL-6 and IL-8 protein expression. Significant changes reported ^*^p < 0.05, ^**^p < 0.001;^***^p< 0.0005 at Student’s *t*-test.

To further investigate CRBPI-related gene signalling, we transfected CRBPI gene in HaCaT cells, an immortalized human keratinocyte line not-expressing CRBPI and analyzed changes of AKT and NF-kB-related genes (Figure 6). The following genes resulted up-regulated significantly in CBBPI transfected cells: 14-3-3-sigma, M-TOR, AKT1, AKT3, FOXO3, P21, P27, P53, P70, PDK1, BAD, PI3K, P-TEN, CASP9, IKK, COX2, TNFα, FASL, INF-ɣ, INFB1, MDR1 and MMP1. Instead, the following genes were down-regulated: BCL2, IL-2, IL-6, IL-8 MYC1, NOS2, VCAM1. Remaining genes analysed were not significantly modified. Blot analysis for IL6, IL8 and pAKT and pERK (p42-44) gave similar results (Figure 6). Those findings strongly supported the hypothesis that CRBPI presence potentially increased some of AKT and NF-κB-related gene and protein expression, probably for the activation in HaCaT cells of FABP5 and PPRβ/δ receptors [19]. Interestingly, in presence of CRBPI some of pro-inflammatory genes were down-regulated, as IL-6 that induces activation of Th cells and controls the balance between Treg cells and Th17 cells [20, 21].

### *RBPI gene* analysis in psoriatic patients

To investigate the potential role of *RBPI* as susceptibility gene, biostatistical analysis was performed in a large cohort of psoriatic (n = 400) and control subjects (n = 490). As reported in haplotype analysis (Supplementary Tables 1 and 2), genetic association and allele/genotype frequencies of the selected SNPs did not statistically vary (*p*>0.05). Haplotype analysis performed on the SNPs of interest revealed several haplotypes combinations. As reported in Supplementary Table 2, the identified haplotypes failed to show a significant association between psoriatic and tested SNPs, at least in our cohort of patients.

## DISCUSSION

In our study, we documented the increased CRBPI expression in human skin psoriatic lesions and the greater severity of lesions in CRBPI-knockout mice in an imiquimod-induced model of psoriasis. CRBPI is a small cytosolic 15KDa protein widely expressed and evolutionarily conserved in many tissues [7]. CRBPI acts as chaperone protein and regulates uptake and subsequent esterification of retinol and its bioavailability [9, 10]. Topical or systemic retinoid treatment contributes to the management of patients with skin psoriasis [22]. The increased CRBPI expression in human psoriatic epidermis confirms previous studies [12], and likely favours the keratinocyte conversion of retinol in retinoic acid, the most active retinoid in the skin [12]. It has been also reported that CRBPI expression in epidermis is up-regulated after the exposure to retinoids and retinol [23–25], suggesting a key role in their efficacy. Retinoids effects are likely related to the transcription of genes involved in cell proliferation and differentiation [25, 26]. Although CRBPI deficiency has no apparent consequence on development and post-natal life, CRBPI-mediated retinoid bioavailability is likely to sustain proliferation and differentiation of keratinocytes [8, 27, 28]. Psoriasis is a hyperproliferative epidermal disorder characterized by spinous layer hyperplasia and incomplete differentiation of granular and cornified layers [29]. Keratinocyte cycle time is reduced approximately eight fold in psoriatic compared to normal skin and the number of dividing cells is greatly increased, resulting in a hyperplastic epithelium [30]. Abnormal proliferation is also confirmed by the increased suprabasal Ki-67 positivity, otherwise observed only in the basal layer of normal epidermis [29, 31]. In addition, an aberrant keratinocyte differentiation for the accelerated turnover also occurs in psoriatic epidermis [4]. CK1 and CK17 are markers to evaluate keratinocyte differentiation and CK17 reduction considered an index of efficacy of anti-psoriatic treatment [21, 32]. To better investigate the role of CRBPI in psoriasis, we induced skin lesions by imiquimod in mice, a diffuse experimental model of psoriasis [17, 18]. Imiquimod, a ligand for TLR7 and TLR8, is a potent immune activator also used for the treatment of virus-associated skin abnormalities as well as (pre)cancerous skin lesions and superficial basal cell carcinomas [33]. Imiquimod-induced psoriasis-like inflammation in the mouse skin recapitulates many clinical and microscopic hallmarks of human psoriasis, [34]. We documented that imiquimod induces more severe skin lesions in CRBPI-knockout compared to wild-type mice. In particular, early after treatment, thickness, hyperkeratosis, erythema, scaling were significantly increased in imiquimod-treated CRBPI-knockout compared with wild-type mice. Also microscopic features of psoriasis, as acanthosis, parakeratosis, papillomatosis and spongiosis [35], increased more in imiquimod-induced lesions of CRBPI-knockout compared to wild-type mice. As concerning skin psoriasis biomarkers, we observed greater Ki-67 and CK17 and lower CK1 expression in imiquimod-induced psoriasis like lesions of CRBPI-knockout compared to wild-type mice. Our results strongly supported the hypothesis that the absence of CRBPI expression favors a more severe phenotype of skin psoriasis. Beside the alteration of psoriasis biomarkers, we documented in imiquimod-treated mouse CRBPI-knockout skin a more evident reduced expression of other retinoid binding proteins, such as CRABPII and RXRα. Activation of EGFR/AKT/ERK pathway has been reported as implicated in the pathogenesis of psoriasis and influencing differentiation, proliferation and apoptosis-related genes expression [36, 37]. A possible interaction between CRBPI alone or homodimerized/heterodimerized with RARα or RARα/RXRα and the consequent modulation of EGFR/AKT/ERK pathways were previously suggested [38]. We documented in imiquimod-treated CRBPI-knockout mice the increase of AKTs, MAPKs and IL-2 and the down-regulation of EGR1 gene level compared with imiquimod-treated wild-type skin. It is possible to speculate that in psoriatic epidermis a reduced or absent CRBPI expression on alternative activation or up-regulation of FOS or MAPK family genes [21, 39]. EGR1 overexpression has been identified in psoriasis and considered a gene involving in the control of benign keratinocyte hyperproloferation [40]. To further confirm the crucial role of CRBPI in maintaining keratinocyte differentiation, inflammatory balance and homeostasis of epidermis, we investigated the effects of CRBPI transfection in HaCaT cells, an immortalized human keratinocytes line, an useful *in vitro* model to evaluate keratinocyte gene modulation [41]. We documented in CRBPI-transfected HaCaT cells the increased expression of several transcriptional NF-κB and AKT-related genes and phosphorylated proteins. Those transcriptional pathways play a key role to sustain keratinocyte proliferation and the accelerated differentiation in psoriatic epidermis [42]. Our data are in accordance with previous studies, in other cell lines. CRBPI transfection in A549 lung cancer cells up-regulated proliferative and transcriptional genes, including pAKT, pEGFR, pErk1/2, creb1 and c-jun [38]; among these, FABP5 and PPRβ/δ trigger inflammatory changes and the deregulation of proliferation [19, 43]. Surprisingly, we documented in CRBPI-transfected HaCaT the down-regulation of some of IL-related genes and proteins, as IL-2, IL-6, and IL-8. In a recent meta-analysis [20, 44], ILs and other cytokines has been suggested as potential biomarkers for psoriasis and for the response to treatment. IL-2, IL-6 are proinflammatory cytokines that induce the activation of Th cells and control the balance between Treg cells and Th17 cells [21]. We also investigated the possible role of CRBPI as susceptibility psoriatic gene (*RBPI gene*). *RBPI* maps on chromosome 3q2 and clusters near to PSORS5 locus [16, 45] a well-known psoriasis susceptibility locus. We analyzed three different SNPs and seven different haplotypes, but no significant associations could be highlighted; although other SNPs on CRBPI gene potentially associated with the disease should be further investigated, at least for the population of Central Italy we considered.

In conclusion, our results strongly support the main involvement of CRBPI in the pathobiology of psoriatic skin. In particular, increased of CRBPI expression seems to be required to sustain accumulated proliferation and abnormal differentiation of keratinocytes. Increased CRBPI expression in psoriatic epidermis may reflect the enhanced necessity of keratinocytes to accumulate retinol to sustain abnormal proliferation and transcriptional activity [39]. Finally, a direct role of CRBPI in counteracting activation of pro-inflammatory IL-related genes could be hypothesized. Strategies aimed to prevent loss of CRBPI expression and its related activity may represent an additional innovative vision aimed to increase efficacy of local and systemic retinoid-based therapies of psoriasis.

## MATERIALS AND METHODS

### Study population and skin biopsies

Fifty-nine patients (19 female and 40 male patients, mean age 47.47±12.1 yrs), affected by moderate-to-severe plaque-type skin psoriasis were consecutively enrolled in an open, observational study at the Dermatology Departments of Tor Vergata University of Rome and S. Gallicano Institute of Rome, Italy [20]. Demographic and clinic-pathological data reported in Table 1. Approval of the study obtained from institutional Ethical Committees and Public Health Ministry in accordance with the Declaration of Helsinki. An age- and sex- matched control group included 20 healthy untreated subjects undergoing an Institutional health surveillance program. Three mm punch biopsies obtained from lesional and non-lesional areas for morphological confirmation of the disease (see after). As control, homogeneous by gender and age, anonymous control skin sections from paraffin block archive of surgically excided non-neoplastic biopsies were also investigated (n=13; mean age 45.5±2.75; range 24-58).

**Table 1:** Demographics and disease parameters of enrolled patients

Case (^*^)	Age	Sex	Pasi	Arthritis
1	39	F	13,5	YES
2	44	M	6	NO
3	24	F	12,9	YES
4	41	M	12,4	NO
5	50	F	15	YES
6	51	M	12	NO
7	54	M	12	NO
8	33	M	13,5	NO
9	55	F	34,2	YES
10	28	M	10	NO
11	31	M	10	NO
12	66	M	13	YES
13	49	M	15,3	YES
14	53	F	12	YES
15	60	M	8,7	YES
16	48	M	12,4	NO
17	32	M	10	NO
18	44	M	15	YES
19	42	M	7	YES
20	57	M	9	YES
21	60	F	10	NO
22	73	M	10	YES
23	57	F	8	YES
24	43	M	13	YES
25	42	M	16	NO
26	42	M	12	YES
27	49	M	10	NO
28	31	M	10	NO
29	39	M	12	NO
30	28	M	12	NO
31	36	F	12	YES
32	54	M	7	YES
33	45	F	4	YES
34	64	F	6	YES
35	35	F	4	YES
36	42	M	12	NO
37	44	M	12	NO
38	29	M	15	YES
39	63	F	12	NO
40	56	M	10	NO
41	46	M	5	YES
42	68	F	1,8	YES
43	57	M	8,5	NO
44	61	F	3,2	YES
45	36	M	33	NO
46	46	M	10,8	NO
47	67	F	6,7	YES
48	70	M	27,9	NO
49	53	M	9,8	NO
50	32	M	15,6	NO
51	49	F	5,6	YES
52	54	M	10,3	NO
53	35	M	12,2	NO
54	56	M	10,4	NO
55	58	F	18,2	NO
56	21	M	10,8	NO
57	56	M	26	NO
58	49	F	5,6	YES
59	54	F	39,6	NO

(^*^) Cases1-40: patients from Policlinico of Tor Vergata.

Cases 41-59. patients from Instituto San Gallicano, Rome, Italy

### Microscopic and immunohistochemical studies of human skin

Biopsies were fixed in 10% formalin for 24 h and paraffin-embedded four-μm thick serial sections stained with Haematoxylin&Eosin for routine histopathological examination or employed for immunohistochemistry. For the latter, sections were incubated with following primary antibodies: polyclonal rabbit anti-CRBPI (1:300; Santa Cruz Biotechnology, Texas, USA); anti-CRABPII (1:300; Bethyl Laboratories, Texas, USA); anti-RXRα (1:750; Abcam; UK) and anti-CK1(1:1000; Abcam) followed by biotin-labeled goat anti-rabbit secondary antibody and streptavidin-horseradish peroxidase conjugated (Ylem,1:100). Serial slides were also incubated with monoclonal mouse anti-CK17 (1:50; Cell Marque Corporation, CA, USA), followed by revelation with Ultravision LP Detection System (Thermo Scientific, CA, USA) [46]. Immunostaining with anti-Ki-67 mouse monoclonal antibody (clone 30-9), performed by using an automatic device (Ventana-Roche Diagnostics Milan, Italy), according to manufacturer’s instructions. All immunohistochemical procedures included positive and negative controls [47].

### Generation of CRBPI-null mice and mutant genotyping

Transgenic C57BL/6 mice heterozygous for *CrbpI gene* (50% 129Sv and 50% C57BL/6, line QK10) were gifted (dr Ghyselinck NB; France). To confirm homozygous CRBPI mutant mice progenies, tail tissue DNA was obtained using manufacture’s extraction protocols (Qiagen, Hilden-Germany). The genotype was confirmed by reverse transcription PCR analysis, as reported [39]. Mice were kept in pathogen-free conditions and housed in a temperature-controlled facility with a 12 light/dark cycle. Animals fed with a commercial diet and water ad libitum. All experiments were in accordance with the ethical standards, the Declaration of Helsinki and with national and international guidelines. All experiments were approved by the Institutional Animal Care and Use Committee, and carried out according to the Italian rules (D.L. 116/92; CE. 609/86).

### Imiquimod-induced psoriasis and monitoring of lesions

Twelve week-old CRBPI-knockout (n=8) and wild-type mice (n=8) were shaved on their back and then treated with a daily topical dose of 62.5 mg of a commercial cream containing 5% imiquimod (Aldara™, MEDA Pharma) for 6 consecutive days [17]. Control CRBPI-knockout and wild type mice (n=3) were treated similarly with a control vehicle cream (Vaseline Bianca, Polifarma Benessere). Imiquimod-treated mice received also the injection of 300 μl of PBS at day 3 and 4, to prevent imiquimod treatment induced systemic adverse effects in C57BL/6 mice [17]. To evaluate the severity of skin lesions, we used a scoring system based on the clinical Psoriasis Area and Severity Index (PASI), consisting of the following parameters: erythema, scaling and skin thickness. Those parameters were scored independently (scale from 0 to 4: 0, none; 1, slight; 2, moderate; 3, marked and 4, very marked) and cumulatively as PASI [17, 18].

### Microscopic and immunohistochemical analysis of mice skin

After 6 days, mice were sacrificed by cervical dislocation and dorsal lesional skin was collected for histological examination. Formalin-fixed, paraffin-embedded sections from skin samples were stained with Haematoxylin&Eosin and examined in a blinded manner by two dermatopathologists. Histopathologic features of psoriasis [35], as hyperkeratosis, parakeratosis, papillomatosis, spongiosis, microabscesses neutrophils and dilated dermal blood vessels were evaluated by using a grading system in arbitrary units as follows: absent presence (0), weak (1), moderate (2), strong (3). Presence of hypogranulosis, acanthosis (as number of epidermal layers in at least ten randomly selected fields) were also evaluated [35].

For immunohistochemistry, serial 4-μm thick mouse skin sections were deparaffinized, rehydrated and, after antigen retrieval and nonspecific peroxidase blocking, incubated with rabbit polyclonal anti-CRBP-I, anti-CRABP-II, anti-RXRɑ and anti-CK1 (see Human immunohistochemistry section), rabbit monoclonal anti-Ki-67 (Novus Biologicals, Littleton, CO, USA) and anti-CK17 (ProteinGroupTech, Manchester, United Kingdom). Positive and negative controls were included to confirm the consistency of the analysis. Semiquantitative evaluation of investigated parameters was performed independently from two researchers by using an arbitrary grading system, as reported [48].

### RNA extraction and gene analysis of mouse skin

Total RNA from the mouse back skin was extracted using TRI^®^ reagent (Sigma-Aldrich, St. Louis, USA) according to the manufacturer’s instructions [49]. RT^2^ profiler PCR arrays (Qiagen, Hilden, Germany) for a gene panel of mouse (PAMM-040ZA; Supplementary Table 3) epidermal growth factor and platelet-derived growth factor-related pathways were performed and data analyzed using the manufacturer’s integrated web-based software package using ΔΔC_t_-based fold-change calculation (Qiagen) in triplicate experiments.

### Cell transfection

Immortalized human keratinocyte cells HaCaT, (Lonza, Milano, Italy) maintained in D-MEM (Lonza Bio Pharma AG, Switzerland) were transfected by using a vector pTargeT Mammalian expression system carrying the whole sequence of CRBPI gene (NM_002899) and the gene for G418 resistance (Promega, Italy), or the G418-resistance gene alone. After 20 days, stable transfected clones were collected in G418-containing medium and tested by PCR and western blot. The correct plasmid sequence was confirmed by Sanger sequencing. Experimental procedures were repeated by using different transfected clones, which gave similar results.

### Gene and protein expression analysis in HaCaT cells

Gene expression analysis of HaCaT cells was performed using Signosis array (Signosis, Inc. Santa Clara, CA, USA). Briefly, RNA was extracted, reverse-transcribed into cDNA in the presence of biotin-dUTP and a profile of 24 genes for human AKT and NF-κB pathway cDNA plate array (Signosis) respectively, was carried out through the detection of streptavidin-HRP. Luminescence reported as relative light units (RLUs) on a microplate luminometer, according to the manufacturer’ instruction. Experiments performed in triplicate. For western blot analyses, after extraction, proteins were blotted onto nitrocellulose membranes and incubated with anti-phosphorylated v-akt murine thymoma viral oncogene homolog (pAkt Ser^473^), anti-AKT (pan), anti-phosphorylated extracellular-signal-regulated kinases (pErk1/2; Cell Signaling Technology, Danvers, MA, USA), anti-interleukin 6 (IL-6, R&D Systems, Minneapolis, USA), anti-interleukin 8 (IL-8, Abcam, Cambridge, UK) and anti-total tubulin antibody (Sigma-Aldrich). Revelation and densitometric blot analysis were performed in three independent experiments. Membranes were reblotted with anti-tubulin to ensure equal loading. Akt activity was expressed as phospho/total protein ratio. Results are mean values ± SEM of the three different experiments. ^*^p < 0.05, ^**^p < 0.005 and ^***^p <0,001. Abbreviations: ADU, arbitrary densitometric units.

### Genetic analysis

The CRBPI gene analysis, were enrolled 890 Italian subjects including patients with psoriasis (n=400) and healthy subjects (n=490). Psoriasic patients were recruited from the Department of Dermatology “Tor Vergata” University of Rome, IDI-Istituto Dermopatico dell’Immacolata, Fatebenefratelli and San Gallicano Hospital, Rome, Italy. The control group were recruited in a wide cohort of blood donors originating from the same geographical area of the cases. The study was approved by the ethics committee of each participating institute and the signature of the informed consent was obtained from all the participants. Genomic DNA was extracted from peripheral blood using standard procedures. The International HapMap Project “*HapMap3 Genome Browser release #2 (Phase 3-genotypes, frequencies & LD)*” has been used to perform a “*in silico*” SNPs selection, in order to identify the fewest number of SNPs able to represent the maximum variability of the region 3q23. In particular, we have selected 4 different SNPs located on *RBP1* gene (rs184157 A/G; rs295493 A/G; rs893704 G/T and rs2071387 C/T). The genotyping analysis was performed by TaqMan assays on a 7500 Fast Real Time PCR device (Applied Biosystems, CA, USA). Each Real Time PCR run contained a negative control and three positive control samples were previously confirmed by direct sequencing. The genotyping data obtained by Real Time PCR were interpreted by using the Sequence Detection System 2.1 software (Applied Biosystems).

### Statistical analysis

All results were expressed as the arithmetical mean±SEM. Data were analyzed by one-way analysis of variance (ANOVA) followed from a Bonferroni post hoc test and using the Student *t*-test. The differences were considered statistically significant for p values <0.05. Statistical genetic analysis was performed by standard 2x2 table and Fisher’s exact text, considering allele, genotype and haplotype frequencies of all tested variations. Odds Ratios (OR) were calculated and allele and haplotype analysis in unrelated samples by using the UNPHASED software [3].

## SUPPLEMENTARY MATERIALS FIGURE AND TABLES




