# Supplementary material for: Expression and potential role of cellular retinol binding protein I in psoriasis
Source: Oncotarget. 2018 Dec 4;9(95):36736–49. doi: 10.18632/oncotarget.26314 (PMC6298411; doi:10.18632/oncotarget.26314)
Supplement: Supplementary file 1 [file oncotarget-09-36736-s001.pdf]

Expression and potential role of cellular retinol binding protein I in psoriasis

SUPPLEMENTARY MATERIALS

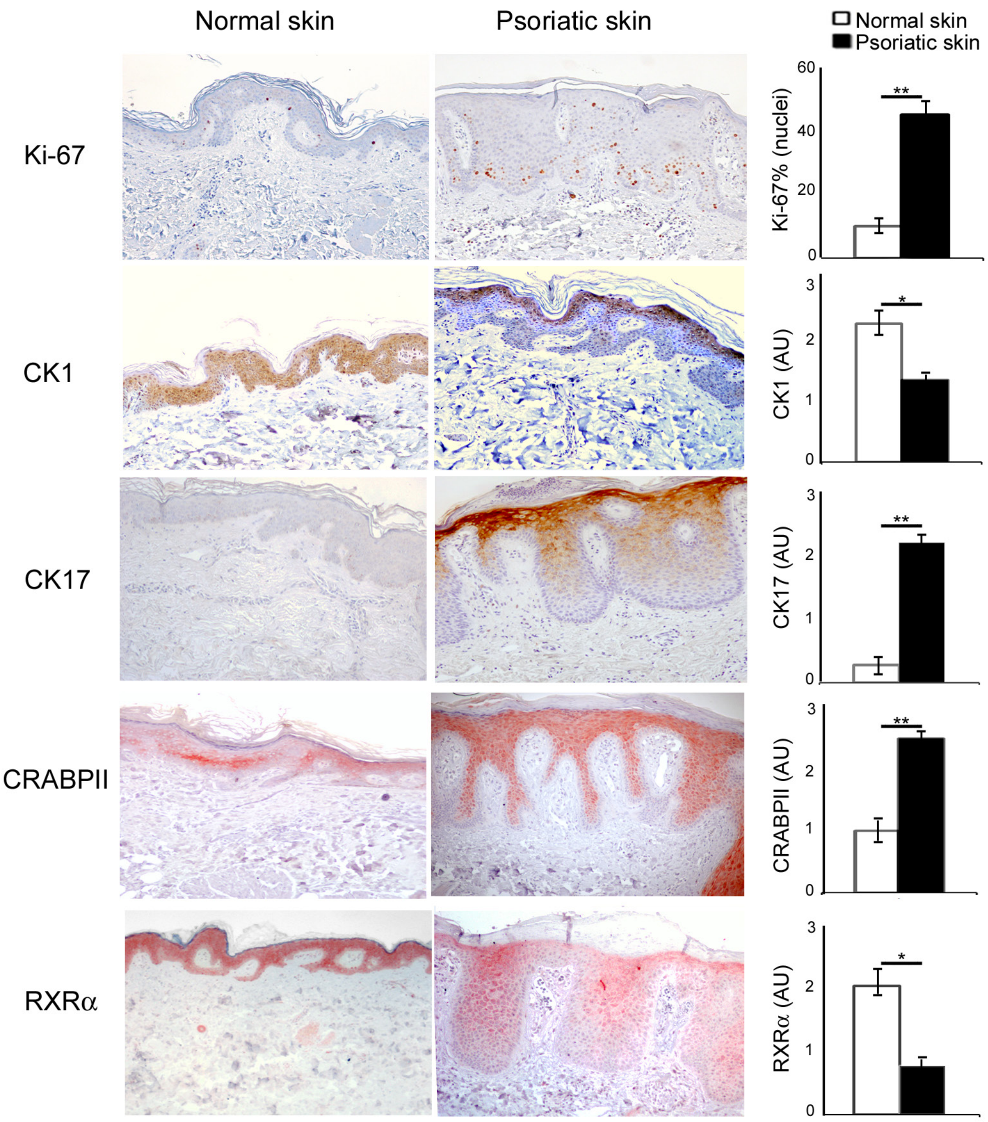

Supplementary Figure 1: Expression of Ki-67, CK1, CK17, CRBPII and RXRα in human normal and psoriatic skin. Left panel: Representative immunostaining showing an increase of epidermal Ki-67 positive nuclei, CK17 and CRABPII expression in psoriatic skin; whereas RXRα and CK1 expression decrease. Original magnification, 100X. $^{*}p < 0.05$; $^{**}p < 0.01$ at Student's *t*-test. Right panel: Bar graphs showing the percentages of markers expression in normal and psoriatic skin.

Supplementary Table 1: Biostatistical analysis of selected CRBPI SNPs

Selected SNPs							
**rs184157 A/G**		**rs295493 A/G**		**rs893704 G/T**		**rs2071387 C/T**	
**Allele Frequency**	***p***	**Allele Frequency**	***p***	**Allele Frequency**	***p***	**Allele Frequency**	***p***
**Cases**		**Cases**		**Cases**		**Cases**	
A: 0.224		A: 0.37		G: 0.43		C: 0.23	
G: 0.776		G: 0. 63		T: 0. 57		T: 0. 77	
**Controls**	ns	**Controls**	ns	**Controls**	ns	**Controls**	ns
A: 0.224		A: 0.36		G: 0.40		C: 0.20	
G: 0.776		G: 0.64		T: 0.60		T: 0.80	
**Genotype Frequency**	***p***	**Genotype Frequency**	***p***	**Genotype Frequency**	***p***	**Genotype Frequency**	***p***
**Cases**		**Cases**		**Cases**		**Cases**	
AA: 0.04		AA: 0.15		GG: 0.17		CC: 0.04	
AG: 0.36		AG: 0.45		GT: 0.51		CT: 0.36	
GG: 0.60		GG: 0.40		TT: 0.32		TT: 0.60	
**Controls**	ns	**Controls**	ns	**Controls**	ns	**Controls**	ns
AA: 0.04		AA: 0.12		GG: 0.18		CC: 0.04	
AG: 0.38		AG: 0.48		GT: 0.44		CT: 0.31	
GG: 0.58		GG: 0.40		TT: 0.38		TT: 0.65	

Supplementary Table 2: Haplotype analysis

Haplotypes	Psv patients Frequencies	Control subjects Frequencies	*p*
G-A-G-C	0.036	0.027	
G-A-G-T	0.154	0.142	
G-A-T-C	0.003	0	
G-A-T-T	0.008	0.007	
G-G-G-C	0.097	0.106	
G-G-G-T	0.014	0.004	
G-G-T-C	0.013	0	
G-G-T-T	0.45	0.4873	ns
A-A-G-C	0.03	0.016	
A-A-G-T	0.08	0.101	
A-A-T-C	0.033	0.032	
A-A-T-T	0.02	0.022	
A-G-G-C	0.002	0.003	
A-A-G-T	0.002	0	
A-G-T-C	0.008	0	
A-G-T-T	0.03	0.04995	

Supplementary Table 3: Analyzed genes array.

See Supplemenatary File 1
