# Supplementary material for: Expression and potential role of cellular retinol binding protein I in psoriasis
Source: Oncotarget. 2018 Dec 4;9(95):36736–49. doi: 10.18632/oncotarget.26314 (PMC6298411; doi:10.18632/oncotarget.26314)
Supplement: Supplementary file 2 [file oncotarget-09-36736-s002.docx]

Table S3. Analyzed genes array

|  |  |  |  |
| --- | --- | --- | --- |
| **Unigene** | **Refseq** | **Symbol** | **Description** |
| Mm.259045 | NM_146243 | Actr2 | ARP2 actin-related protein 2 homolog (yeast) |
| Mm.6645 | NM_009652 | Akt1 | Thymoma viral proto-oncogene 1 |
| Mm.177194 | NM_007434 | Akt2 | Thymoma viral proto-oncogene 2 |
| Mm.235194 | NM_011785 | Akt3 | Thymoma viral proto-oncogene 3 |
| Mm.220946 | NM_009703 | Araf | V-raf murine sarcoma 3611 viral oncogene homolog |
| Mm.676 | NM_007497 | Atf1 | Activating transcription factor 1 |
| Mm.209903 | NM_009715 | Atf2 | Activating transcription factor 2 |
| Mm.4387 | NM_007522 | Bad | BCL2-associated agonist of cell death |
| Mm.3758 | NM_009954 | Bcar1 | Breast cancer anti-estrogen resistance 1 |
| Mm.257460 | NM_009741 | Bcl2 | B-cell leukemia/lymphoma 2 |
| Mm.245513 | NM_139294 | Braf | Braf transforming gene |
| Mm.34405 | NM_009810 | Casp3 | Caspase 3 |
| Mm.88829 | NM_015733 | Casp9 | Caspase 9 |
| Mm.328206 | NM_001033238 | Cblb | Casitas B-lineage lymphoma b |
| Mm.273049 | NM_007631 | Ccnd1 | Cyclin D1 |
| Mm.3996 | NM_007700 | Chuk | Conserved helix-loop-helix ubiquitous kinase |
| Mm.277735 | NM_007742 | Col1a1 | Collagen, type I, alpha 1 |
| Mm.422634 | NM_133828 | Creb1 | CAMP responsive element binding protein 1 |
| Mm.23692 | NM_007788 | Csnk2a1 | Casein kinase 2, alpha 1 polypeptide |
| Mm.378901 | NM_009975 | Csnk2b | Casein kinase 2, beta polypeptide |
| Mm.239041 | NM_013642 | Dusp1 | Dual specificity phosphatase 1 |
| Mm.1791 | NM_026268 | Dusp6 | Dual specificity phosphatase 6 |
| Mm.252481 | NM_010113 | Egf | Epidermal growth factor |
| Mm.439882 | NM_007912 | Egfr | Epidermal growth factor receptor |
| Mm.181959 | NM_007913 | Egr1 | Early growth response 1 |
| Mm.3941 | NM_007917 | Eif4e | Eukaryotic translation initiation factor 4E |
| Mm.235346 | NM_007945 | Eps8 | Epidermal growth factor receptor pathway substrate 8 |
| Mm.3355 | NM_010177 | Fasl | Fas ligand (TNF superfamily, member 6) |
| Mm.193099 | NM_010233 | Fn1 | Fibronectin 1 |
| Mm.246513 | NM_010234 | Fos | FBJ osteosarcoma oncogene |
| Mm.338613 | NM_019740 | Foxo3 | Forkhead box O3 |
| Mm.277409 | NM_021356 | Gab1 | Growth factor receptor bound protein 2-associated protein 1 |
| Mm.439649 | NM_008163 | Grb2 | Growth factor receptor bound protein 2 |
| Mm.491101 | NM_001031667 | Gsk3a | Glycogen synthase kinase 3 alpha |
| Mm.394930 | NM_019827 | Gsk3b | Glycogen synthase kinase 3 beta |
| Mm.289681 | NM_010415 | Hbegf | Heparin-binding EGF-like growth factor |
| Mm.334313 | NM_008284 | Hras | Harvey rat sarcoma virus oncogene 1 |
| Mm.277886 | NM_010546 | Ikbkb | Inhibitor of kappaB kinase beta |
| Mm.14190 | NM_008366 | Il2 | Interleukin 2 |
| Mm.289657 | NM_146145 | Jak1 | Janus kinase 1 |
| Mm.275071 | NM_010591 | Jun | Jun oncogene |
| Mm.399068 | NM_001031811 | Kcnh8 | Potassium voltage-gated channel, subfamily H (eag-related) |
| Mm.383182 | NM_021284 | Kras | V-Ki-ras2 Kirsten rat sarcoma viral oncogene homolog |
| Mm.87787 | NM_010735 | Lta | Lymphotoxin A |
| Mm.248907 | NM_008927 | Map2k1 | Mitogen-activated protein kinase kinase 1 |
| Mm.412922 | NM_009157 | Map2k4 | Mitogen-activated protein kinase kinase 4 |
| Mm.3906 | NM_011944 | Map2k7 | Mitogen-activated protein kinase kinase 7 |
| Mm.211762 | NM_011946 | Map3k2 | Mitogen-activated protein kinase kinase kinase 2 |
| Mm.196581 | NM_011949 | Mapk1 | Mitogen-activated protein kinase 1 |
| Mm.39253 | NM_009158 | Mapk10 | Mitogen-activated protein kinase 10 |
| Mm.8385 | NM_011952 | Mapk3 | Mitogen-activated protein kinase 3 |
| Mm.21495 | NM_016700 | Mapk8 | Mitogen-activated protein kinase 8 |
| Mm.68933 | NM_016961 | Mapk9 | Mitogen-activated protein kinase 9 |
| Mm.209327 | NM_021461 | Mknk1 | MAP kinase-interacting serine/threonine kinase 1 |
| Mm.4825 | NM_010810 | Mmp7 | Matrix metallopeptidase 7 |
| Mm.389903 | NM_010879 | Nck2 | Non-catalytic region of tyrosine kinase adaptor protein 2 |
| Mm.383185 | NM_010901 | Nfatc3 | Nuclear factor of activated T-cells, cytoplasmic, |
| Mm.256765 | NM_008689 | Nfkb1 | Nuclear factor of k light polypeptide gene enhancer B-cells 1 |
| Mm.400954 | NM_010937 | Nras | Neuroblastoma ras oncogene |
| Mm.2565 | NM_053074 | Nup62 | Nucleoporin 62 |
| Mm.2675 | NM_008808 | Pdgfa | Platelet derived growth factor, alpha |
| Mm.144089 | NM_011057 | Pdgfb | Platelet derived growth factor, B polypeptide |
| Mm.221403 | NM_011058 | Pdgfra | Platelet derived growth factor receptor, alpha polypeptide |
| Mm.10504 | NM_011062 | Pdpk1 | 3-phosphoinositide dependent protein kinase 1 |
| Mm.260521 | NM_008839 | Pik3ca | Phosphatidylinositol 3-kinase, catalytic, alpha polypeptide |
| Mm.259333 | NM_001024955 | Pik3r1 | Phosphatidylinositol 3-kinase, regulatory subunit 1 |
| Mm.12945 | NM_008841 | Pik3r2 | Phosphatidylinositol 3-kinase, regulatory subunit, 2 |
| Mm.154660 | NM_008872 | Plat | Plasminogen activator, tissue |
| Mm.44463 | NM_021280 | Plcg1 | Phospholipase C, gamma 1 |
| Mm.260288 | NM_019411 | Ppp2ca | Protein phosphatase 2 (formerly 2A), catalytic subunit, |
| Mm.222178 | NM_011101 | Prkca | Protein kinase C, alpha |
| Mm.245395 | NM_008960 | Pten | Phosphatase and tensin homolog |
| Mm.184163 | NM_029780 | Raf1 | V-raf-leukemia viral oncogene 1 |
| Mm.365961 | NM_145541 | Rap1a | RAS-related protein-1a |
| Mm.259653 | NM_145452 | Rasa1 | RAS p21 protein activator 1 |
| Mm.757 | NM_016802 | Rhoa | Ras homolog gene family, member A |
| Mm.220417 | NM_153587 | Rps6ka5 | Ribosomal protein S6 kinase, polypeptide 5 |
| Mm.394280 | NM_028259 | Rps6kb1 | Ribosomal protein S6 kinase, polypeptide 1 |
| Mm.86595 | NM_011368 | Shc1 | Src homology 2 domain-containing transforming protein C1 |
| Mm.22845 | NM_009271 | Src | Rous sarcoma oncogene |
| Mm.277406 | NM_009283 | Stat1 | Signal transducer and activator of transcription 1 |
| Mm.473190 | NM_011486 | Stat3 | Signal transducer and activator of transcription 3 |
| Mm.277403 | NM_011488 | Stat5a | Signal transducer and activator of transcription 5A |
| Mm.222 | NM_011640 | Trp53 | Transformation related protein 53 |
| Mm.328431 | NM_007393 | Actb | Actin, beta |
| Mm.163 | NM_009735 | B2m | Beta-2 microglobulin |
| Mm.309092 | NM_008084 | Gapdh | Glyceraldehyde-3-phosphate dehydrogenase |
| Mm.3317 | NM_010368 | Gusb | Glucuronidase, beta |
| Mm.2180 | NM_008302 | Hsp90ab1 | Heat shock protein 90 alpha (cytosolic), class B member 1 |
| N/A | SA_00106 | MGDC | Mouse Genomic DNA Contamination |
| N/A | SA_00104 | RTC | Reverse Transcription Control |
| N/A | SA_00104 | RTC | Reverse Transcription Control |
| N/A | SA_00104 | RTC | Reverse Transcription Control |
| N/A | SA_00103 | PPC | Positive PCR Control |
| N/A | SA_00103 | PPC | Positive PCR Control |
| N/A | SA_00103 | PPC | Positive PCR Control |
|  |  |  |  |
|  |  |  |  |
|  |  |  |  |
|  |  |  |  |
